# The Instrumental Activities of Daily Living in Parkinson’s Disease Patients Treated by Subthalamic Deep Brain Stimulation

**DOI:** 10.3389/fnagi.2022.886491

**Published:** 2022-06-17

**Authors:** Ondrej Bezdicek, Josef Mana, Filip Růžička, Filip Havlik, Anna Fečíková, Tereza Uhrová, Evžen Růžička, Dušan Urgošík, Robert Jech

**Affiliations:** ^1^Department of Neurology and Centre of Clinical Neuroscience, First Faculty of Medicine and General University Hospital in Prague, Charles University, Prague, Czechia; ^2^Department of Stereotactic and Radiation Neurosurgery, Na Homolce Hospital, Prague, Czechia

**Keywords:** activities of daily living, deep brain stimulation, cognition, everyday abilities, subthalamic nucleus

## Abstract

**Background:**

Everyday functioning and instrumental activities of daily living (IADL) play a vital role in preserving the quality of life in patients with Parkinson’s disease (PD) after deep brain stimulation of the subthalamic nucleus (STN-DBS).

**Objective:**

The main goal of the current study was to examine IADL change in pre-and post-surgery of the STN-DBS. We also analyzed the influence of the levodopa equivalent daily dose (LEDD) and global cognitive performance (Dementia Rating Scale; DRS-2) as covariates in relation to IADL.

**Methods:**

Thirty-two non-demented PD patients were administered before and after STN-DBS neurosurgery the Penn Parkinson’s Daily Activities Questionnaire (PDAQ; self-report), the DRS-2 and Beck Depression Inventory (BDI-II) to assess IADL change, global cognition, and depression.

**Results:**

We found a positive effect of STN-DBS on IADL in the post-surgery phase. Moreover, lower global cognition and lower LEDD are predictive of lower IADL in both pre-surgery and post-surgery examinations.

**Summary/Conclusion:**

STN-DBS in PD is a safe method for improvement of everyday functioning and IADL. In the post-surgery phase, we show a relation of IADL to the severity of cognitive impairment in PD and to LEDD.

## Highlights

- There is a significant effect of STN-DBS treatment on everyday functioning improvement 1 year after the surgery in Parkinson’s disease patients.
- We found also positive effects of cognitive performance, and LEDD as well as a negative effect of depressive symptoms on everyday functioning both before and after STN-DBS surgery.
- We provide a table detailing what changes can one expect in a patient’s everyday functioning depending on their pre- and post-surgery LEDD.

## Introduction

Deficits in everyday functioning are highly associated with the evolution of Parkinson’s disease (PD) (Young et al., 2010; Giovannetti et al., 2012; Altieri et al., 2021; Becker et al., 2022). Especially in clinically advanced stages when patients are suffering from deteriorating cognitive impairment such as mild cognitive impairment (PD-MCI) and later from dementia due to PD (PDD), instrumental activities of daily living (IADL) such as meal preparation, shopping, and medication management are afflicted (Pirogovsky et al., 2014; Foster and Doty, 2021). A standardized assessment of IADL can be specific for the diagnosis of PDD (Christ et al., 2013). Those PD-MCI patients who show more impaired cognitive- than motor-driven IADL have a higher hazard of conversion to PDD (Becker et al., 2020). Regarding PD motor dysfunction subtypes, *de novo* PD patients with postural instability-gait difficulties motor subtype present on average larger deterioration in IADL than those with tremor dominant subtype (Hariz and Forsgren, 2011).

Deep brain stimulation (DBS) of the subthalamic nucleus (STN-DBS) is a standard treatment for medication-refractory movement symptoms of PD (Perlmutter and Mink, 2006; Bronstein et al., 2011; Okun, 2014; Mueller et al., 2020). A number of works have shown that this therapy is highly effective in regaining control over PD motor symptoms and improving patients’ quality of life (QoL), as well as in reducing the levodopa equivalent daily dose (LEDD) (Tomlinson et al., 2010; Bratsos et al., 2018; Tödt et al., 2022).

Hence DBS is a treatment option for advanced PD when the side effects of dopaminergic treatment are intolerable (Moro and Lang, 2006; Bronstein et al., 2011) and there are the differential effects of levodopa vs. DBS on brain motor activity (Mueller et al., 2020), a relation between LEDD and IADL in PD in pre- and post-surgery should come under closer scrutiny.

The effect on QoL is stable in time in a 1-year perspective and the IADL performance correlates with the improvement in QoL (Gorecka-Mazur et al., 2019). On the contrary, activity limitations are the strongest predictor of QoL (Soh et al., 2013). However, the improvement after 12 months after the surgery was noticeable only in some IADLs, especially in shopping and food preparation (Gorecka-Mazur et al., 2019).

Previous research of IADL in PD patients after STN-DBS used general IADL scales, such as Lawton’s IADL scale, to investigate everyday performance (Gorecka-Mazur et al., 2019) or the Unified Parkinson’s Disease Rating Scale (UPDRS part II) or the Schwab and England Activities of Daily Living (Deuschl et al., 2006; Kleiner-Fisman et al., 2010; Odekerken et al., 2013; Jiang et al., 2015; Tödt et al., 2022). In the current study, we sought to use a disease-specific questionnaire developed to assess IADLs in PD (Brennan et al., 2016a). The Penn Parkinson’s Daily Activities Questionnaire (PDAQ) shows a high discriminant validity between PD with normal cognition in comparison to PD-MCI and PDD (Brennan et al., 2016a).

Cognitive impairment is a core non-motor feature of Parkinson’s disease (PD) and a major source of disability in PDD (Cahn et al., 1998; Bronnick et al., 2006). Especially, impaired global cognition and deficits in attention and visual memory are the most predictive of developing a PDD (Lawson et al., 2021). PD-MCI as a pre-dementia phase and PDD both represent risk factors that are associated with poorer everyday functioning and IADL (Litvan et al., 2012; Martin et al., 2013; Pirogovsky et al., 2013; Pirogovsky et al., 2014; Hoogland et al., 2017; Schmitter-Edgecombe et al., 2021).

Thus, the principal aim of the study was to assess post-surgery change (i.e., decline or improvement) in self-reported IADL in relation to the pre-surgery evaluation. Second, we aimed to outline the relationship of IADL to dopaminergic medication, depressive symptoms and cognitive performance in PD patients treated with STN DBS.

## Materials and Methods

### Participants

Parkinson’s disease patients were recruited from the Movement Disorders Center, Department of Neurology, First Faculty of Medicine and General University Hospital in Prague. All patients were examined by a neurologist specializing in movement disorders and met the UK PD Society Brain Bank criteria (Hughes et al., 1992). All of them were suffering from motor fluctuations and/or disabling dyskinesias and were indicated for treatment with STN DBS (demographic and clinical details in Table 1). Exclusion criteria were as follows: PD dementia according to MDS criteria (Emre et al., 2007), atypical or secondary parkinsonism, severe or moderate depression according to Beck Depression Inventory (BDI-II) and psychiatric evaluation, florid psychotic manifestations (hallucinations or delusions), anticholinergic medications and other medical or neurological conditions potentially resulting in cognitive impairment (e.g., epileptic seizure, tumor, stroke, or head trauma). All PD patients were under dopaminergic therapy (i.e., levodopa, dopamine agonist, or a combination of them), and levodopa’s equivalent daily dose for each patient was calculated before and after surgery (Tomlinson et al., 2010). Bilateral STN DBS implantation was performed as previously described (Jech et al., 2006; Urgosik et al., 2011; Jech et al., 2012). STN DBS parameters are reported in Table 1. A total of 32 PD patients (mean age 55.5 ± 7.8 years pre-surgery, 56% males) participated in the study. Patients were assessed before (4.9 ± 5.6 months) and 1 year after the surgery (12.4 ± 0.9 months). All patients gave their written informed consent for participation. The study was approved by the Ethics Committee of the General University Hospital in Prague, Czechia.

**TABLE 1:** Demographic, clinical, and cognitive characteristics of the sample (*N* = 32).

	Pre-surgery	Post-surgery
Age (years)	55.50 ± 7.78	56.95 ± 7.79
Education (years)	14.20 ± 3.25	–
Sex (males)	18 (56%)	–
Disease duration at surgery (years)	11.37 ± 3.67	–
LEDD (mg)	1819.77 ± 693.73	833.32 ± 498.48
Levodopa test (% response)	58.42 ± 11.79	–
MDS-UPDRS III (medication ON)	18.76 ± 9.13	–
MDS-UPDRS III (medication OFF)	44.12 ± 15.05	–
MDS-UPDRS III (stimulation ON)*	–	26.25 ± 10.00
MDS-UPDRS III (stimulation OFF)*	–	45.16 ± 14.04
PDAQ-15 (range 0–60)	51.34 ± 7.49	52.34 ± 6.35
DRS-2 (range 0–144)	139.28 ± 3.62	139.44 ± 3.33
BDI-II (range 0–63)	10.38 ± 7.20	9.91 ± 6.90
Stimulation parameters		
Current right (mA)	–	2.24 ± 0.55
Current left (mA)	–	2.21 ± 0.60
Pulse duration right (μs)	–	62.81 ± 8.88
Pulse duration left (μs)	–	63.64 ± 9.94
Frequency right (Hz)	–	129.06 ± 18.38
Frequency left (Hz)	–	125.76 ± 11.73

*Post-surgery MDS-UPDRS III testing was done in the OFF mediation condition; μs, microseconds; BDI-II, Beck Depression Rating Scale, second edition; DRS-2, Dementia Rating Scale, second edition; Hz, Hertz; LEDD, levodopa equivalent daily dose; mA, milliamperes; MDS-UPDRS III, Movement Disorder Society Unified Parkinson’s Disease Rating Scale, motor part; PDAQ-15, The Penn Parkinson’s Daily Activities Questionnaire-15. The values are presented in a format mean ± standard deviation or number of observations (percentage from the whole sample).

### Assessments

#### Neuropsychological Examination

All patients underwent a comprehensive and recommended pre-surgery (pre-test) evaluation including neuropsychological, psychiatric, and neurological examinations by a trained movement disorders specialist in each field (Kubu, 2018). The patients were followed up in a post-surgery (post-test) 1 year after the neurosurgery with the identical protocol (mean retest interval 12.4 ± 0.9 months). The pre-surgery neuropsychological assessment was performed with regular dopaminergic therapy (ON medication), in the post-surgery phase, patients were examined in both STN DBS ON with optimal stimulation parameters and the ON medication condition.

The neuropsychological assessment in pre-test–post-test followed the standard Movement Disorder Society neuropsychological battery at Level I for PD-MCI (Litvan et al., 2012; Bezdicek et al., 2016; Bezdicek et al., 2017): the cognitive performance was assessed by Mattis Dementia Rating Scale, second edition (DRS-2) (Jurica et al., 2001; Bezdicek et al., 2015). The IADLs and everyday functioning were measured by the PDAQ self-report (Shulman et al., 2016). The PDAQ brief version is an item-response theory (IRT)-based questionnaire consisting of 15 items, showing very good psychometric properties that were developed specifically for IADL deficits in PD (Brennan et al., 2016a,b). Finally, depressive symptoms were assessed with the Beck Depression Scale, second edition (BDI-II) (Beck et al., 1996; Ciharova et al., 2020).

#### Neurological and Psychiatric Examination

All patients underwent a comprehensive clinical evaluation that included medical history, medication status, and motor status by the Movement Disorders Society Unified Parkinson’s Disease Rating Scale, part three (MDS-UPDRS-III). Scores of patients who underwent the older version of the Unified Parkinson’s Disease Rating Scale (UPDRS-III) were converted to the MDS-UPDRS III scale using the method described by Hentz et al. (2015). All PD patients were treated with dopaminergic therapy, consisting of levodopa, dopamine agonists or a combination of them, and assessed in medication ON. Four days before the patient’s visit, dopamine agonists were substituted with equivalent doses of levodopa. The LEDD was calculated at each assessment time-point according to Tomlinson et al. (2010).

A psychiatric evaluation was done before the surgery to exclude pre-psychotic or florid psychotic symptoms or mood disorders including suicidal thoughts or any other potential risky neuropsychiatric complications after the neurosurgery (Foley et al., 2018).

### Causal Assumptions

**FIGURE 1:**
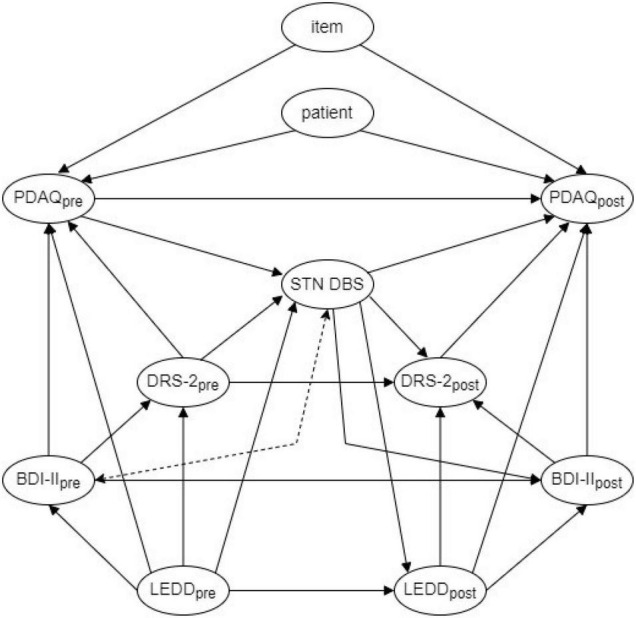
A directed acyclic graph representing causal assumptions of the relationships between included variables. STN DBS, subthalamic nucleus deep brain stimulation; BDI-II, Beck Depression Inventory before DBS treatment (BDI-II_pre_) and after DBS treatment (BDI-II_post_); DRS-2, Dementia Rating Scale, second edition before DBS treatment (DRS-2_pre_) and after DBS treatment (DRS-2_post_); LEDD, levodopa equivalent daily dose before DBS treatment (LEDD_pre_) and after DBS treatment (LEDD_post_); PDAQ, The Penn Parkinson’s Daily Activities Questionnaire before DBS treatment (PDAQ_pre_) and after DBS treatment (PDAQ_post_). STN DBS was considered to be adjusted for in each of our analyses due to the lack of a control group. Dashed double arrow between BDI-II_pre_ and STN DBS indicates a common cause assumption—this is because even though BDI-II is not used directly to decide whether patients receive STN DBS in our center, patients with clinical depression according to an independent psychiatric evaluation are both rejected to STN DBS and at risk of high BDI-II.

Our causal assumptions are represented in the form of a directed acyclic graph (DAG) depicted in Figure 1. Representing study design *via* a DAG offers several benefits including serving as an explicit statement of causal assumptions that can be questioned by other researchers, providing a framework for the interpretation of results and indicating which covariates should be controlled during the analysis (Pearl, 2009; McElreath, 2020). In short, in the current study, we assume that clinicians based their decision on whether to treat a PD patient with STN DBS in part of the patient’s preoperative IADL, objective cognitive performance and LEDD. While the level of depressive symptoms as assessed by BDI-II is not directly considered for STN DBS treatment in our center, patients with the depressive syndrome as assessed by an independent psychiatric evaluation are rejected for STN DBS due to possible suicidal attempts, and since the depressive symptom also likely leads to high BDI-II score, we assume a common cause relationship between pre-surgery BDI-II and STN DBS surgery as indicated by the dashed double-headed arrow in Figure 1. On the other hand, DBS treatment itself is assumed to influence postoperative IADL, objective cognitive performance, level of depressive symptoms and LEDD. Cross-sectionally (i.e., either pre-or post-surgery), LEDD is assumed to influence depressive symptoms directly and objective cognitive performance and IADL both directly as well as indirectly *via* the effect of depressive symptoms. Finally, because IADL was repeatedly assessed by an IRT-based PDAQ questionnaire we expect item- and patient-specific effects on the outcomes.

Since our sample contains only patients treated with STN DBS and no control group, we were not able to estimate either the total or direct effect of DBS on IADL. However, based on the DAG in Figure 1, we can estimate the direct effects of objective cognitive performance, depressive symptoms and LEDD on IADL as well as direct post-surgery change in IADL in STN DBS patients by controlling for LEDD, objective cognitive performance and depressive symptoms as well as item- and patient-specific effects.

### Statistical Analysis

Following the implications of DAG presented in Figure 1, the data were analyzed by a generalized linear mixed model (GLMM) with responses to each item of PDAQ as an outcome, the time of assessment (pre- vs. post-surgery), LEDD, DRS-2, and BDI-II as fixed effects and item- and patient-specific random effects. Interactions of the time of assessment and all LEDD (medication), DRS-2 (cognitive performance), and BDI-II (depressive symptoms) were also included and modeled as fixed effects to explore whether the effect of the latter three variables on IADL changes after as compared to before STN DBS surgery. Since PDAQ consists of 15 items scored by the patient’s self-reported difficulty in performing each specific IADL on a Likert scale ranging from 0 (“cannot do”) to 4 (“no difficulty”), the outcome was modeled by an order-logit response function. The ordered-logit is a generalization of the binary logistic response function that was designed to handle ordinal variables (Liddell and Kruschke, 2018; Bürkner and Vuorre, 2019). The results of an ordered-logit model consist of regression parameters for effect estimates on a logit scale (similar to common logistic regression).

The model was fitted using the Hamiltonian Monte Carlo (HMC) sampling algorithm in Stan version 2.21.0 (Stan Development Team, 2021) accessed *via* brms package (Bürkner and Vuorre, 2019) in R version 4.0.5 (R Core Team, 2021) using four independent chains with 1,500 total and 500 warm-up iterations. Full Bayesian statistical inference was used to specify the model and evaluate the results. We used Student-*t* priors with zero mean, a scale of 2.5 and three degrees of freedom for Intercepts and random effects’ variance components and regularizing Normal priors with zero mean and standard deviation of 0.5 for the fixed effects. GLMM parameters were described on a logit scale by their medians, 95% highest density posterior probability intervals (PPIs) and the probability of being positive (i.e., the probability that a predictor has a positive effect on IADL). A 95% PPI can be interpreted such that a given parameter lies within this interval with a 95% probability. If desired, an effect can be regarded statistically significant (on a 5% level) if the corresponding 95% PPI excludes zero. To evaluate post-surgery change in IADL on the outcome scale we provide posterior predictions comparing the contrast between post-surgery minus pre-surgery responses to PDAQ across patients and items. Scripts with all analyses from this article are deposited here: https://github.com/josefmana/dbs_postop_iADL.

## Results

### Characterizing the Sample

Characteristics of the sample are presented in Table 1. A total of 32 patients with PD and bilateral STN-DBS implanted with DBS devices between 2018 and 2019 met the inclusion criteria. Patients’ responses to each PDAQ item before and after the STN-DBS surgery are depicted in Supplementary Figure 1.

### Results of the Generalized Linear Mixed Model

The HMC sampling algorithm successfully converged to stable posterior distribution (all $\hat{R}$s < 1.01). Fixed effects’ parameters are presented in Table 2. There was a 99.4% probability that patients report improved IADL post- as compared to pre-surgery when covariates are kept constant (the main effect of the time of assessment). Moreover, 95% PPI of this effect excluded zero and the effect can thus be regarded as statistically significant. Similarly, for DRS-2, there was a 98.8% probability that it has a positive main effect on IADL (i.e., higher scores in DRS-2 positively affected IADL regardless of the time of assessment) with 95% PPI excluding zero. On the other hand, while both LEDD and BDI-II showed a trend of the positive main effect on IADL, their 95% PPIs included zero. There was a trend of an interaction between the time of assessment and LEDD, DRS-2, and BDI-II. However, the 95% PPI for these effects included zero as well as moderate negative values and these effects thus cannot be regarded as statistically significant.

**TABLE 2:** Fixed effect parameters of the ordered-logit generalized linear mixed model.

Predictor	*b*	95% PPI	Pr(*b* > 0)
Time of assessment	0.72	[0.21, 1.32]	0.994
LEDD	0.12	[−0.10, 0.35]	0.861
DRS-2	0.31	[0.02, 0.56]	0.988
BDI-II	−0.26	[−0.54, 0.01]	0.031
Time of assessment × LEDD	0.20	[−0.10, 0.51]	0.892
Time of assessment × DRS-2	−0.13	[−0.40, 0.17]	0.188
Time of assessment × BDI-II	−0.14	[−0.48, 0.17]	0.204

*×, statistical interaction; b, median parameter estimate; BDI-II, Beck Depression Inventory; DRS-2, Dementia Rating Scale, second edition; LEDD, levodopa equivalent daily dose; PPI, highest density posterior probability interval; Pr(b > 0), probability that the parameter is positive (i.e., the effect “helps” with IADL evaluated by PDAQ, range 0–1); Time of assessment, pre- vs. post-surgery variable (higher values indicated post-surgery improvement). The time of assessment was deviation coded (i.e., pre-surgery = −0.5, post-surgery = 0.5) such that the main effects of DRS-2 and LEDD reflect the average effects across pre- and post-surgery assessments.*

**FIGURE 2:**
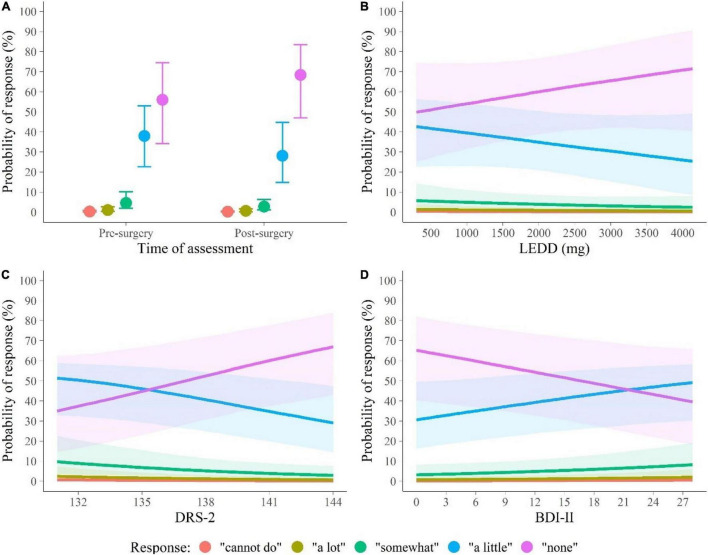
Summary of the marginal distributions of main effects of **(A)** the time of assessment, **(B)** LEDD, **(C)** DRS-2, and **(D)** BDI-II on IADL. BDI-II, Beck Depression Inventory; DRS-2, Dementia Rating Scale, second edition; IADL, instrumental activities of daily living; LEDD, levodopa equivalent daily dose. Points (lines) represent the median probability of each response (labeled by distinct colors) to the items of Penn Parkinson’s Daily Activities Questionnaire-15 (PDAQ-15), and whiskers (shades) represent 95% posterior probability intervals (PPIs).

Figure 2 depicts the main effects of assessment time, LEDD, DRS-2, and BDI-II on responses to PDAQ on the outcome scale (i.e., probabilities that a patient responds with each of the options 0–4). Figure 2 shows that when evaluating difficulties in IADL patients rarely selected options 0–2 (“cannot do,” “a lot,” and “somewhat,” respectively). The main effects of the time of assessment, DRS-2, BDI-II, and LEDD were primarily due to increased frequency of response four (“none”) at the expense of response three (“a little”) after the surgery and in patients with high LEDD and DRS-2 and low BDI-II scores. When LEDD, DRS-2, and BDI-II were statistically held at the average in-sample pre-surgery level, the post-surgery probability that a patient responds to any PDAQ item with option zero (“cannot do”) decreased by 0.1% (95% PPI [−0.4, 0.0], pre: 0.3% [0.1, 0.7], post: 0.1% [0.0, 0.4]), the probability of response one (“a lot”) decreased by 0.4% (95% PPI [−1.2, −0.1], pre: 0.9% [0.3, 2.1], post: 0.5% [0.1, 1.1]), the probability of response two (“somewhat”) decreased by 1.9% (95% PPI [−4.6, −0.3], pre: 4.1% [1.7, 8.4], post: 2.1% [0.6, 4.7]), the probability of response three (“a little”) decreased by 12.3% (95% PPI [−22.2, −3.4], pre: 36.1% [21.8, 51.1], post: 22.9% [10.1, 39.5]), and the probability of response four (“none”) increased by 15.0% (95% PPI [3.1, 26.4], pre: 58.5% [39.2, 77.1], post: 74.4% [55.6, 90.1]). In other words, the direct effect of DBS on IADL improvement is due to a significantly lower frequency of patients with little IADL difficulties (response three “a little” in all PDAQ items) and a significantly higher frequency of patients with no IADL difficulties (response four “none” in all PDAQ items) 1 year after the STN-DBS surgery. This pattern of responses holds across different LEDD levels (see Table 3).

**TABLE 3:** Expected response probabilities of difficulty in IADL stratified by the time of assessment and levodopa equivalent daily dose derived from the ordered-logit GLMM.

Assessment	LEDD (mg)	Pr(resp = 0)	Pr(resp = 1)	Pr(resp = 2)	Pr(resp = 3)	Pr(resp = 4)
Pre-surgery	0	0.5 ± 0.4%	1.7 ± 1.2%	7.0 ± 3.8%	43.1 ± 9.2%	47.6 ± 13.6%
	500	0.4 ± 0.3%	1.5 ± 0.9%	6.1 ± 3.0%	41.5 ± 8.6%	50.4 ± 12.1%
	1,000	0.4 ± 0.3%	1.3 ± 0.7%	5.4 ± 2.4%	39.6 ± 8.0%	53.3 ± 10.9%
	1,500	0.3 ± 0.2%	1.1 ± 0.6%	4.8 ± 2.0%	37.5 ± 7.7%	56.3 ± 10.1%
	2,000	0.3 ± 0.2%	1.0 ± 0.5%	4.3 ± 1.8%	35.3 ± 7.7%	59.1 ± 9.9%
	2,500	0.3 ± 0.2%	0.9 ± 0.5%	3.9 ± 1.8%	33.1 ± 8.1%	61.9 ± 10.2%
	3,000	0.2 ± 0.2%	0.8 ± 0.5%	3.5 ± 1.8%	31.0 ± 8.7%	64.4 ± 10.9%
	3,500	0.2 ± 0.2%	0.8 ± 0.5%	3.2 ± 1.9%	29.0 ± 9.5%	66.8 ± 11.8%
	4,000	0.2 ± 0.2%	0.7 ± 0.5%	3.0 ± 2.0%	27.2 ± 10.4%	68.9 ± 12.8%
	4,500	0.2 ± 0.2%	0.7 ± 0.6%	2.8 ± 2.2%	25.5 ± 11.2%	70.9 ± 13.8%
	5,000	0.2 ± 0.2%	0.6 ± 0.6%	2.7 ± 2.4%	23.9 ± 12.0%	72.6 ± 14.8%
Post-surgery	0	0.5 ± 0.3%	1.6 ± 0.9%	6.7 ± 3.0%	43.2 ± 7.9%	48.0 ± 11.5%
	500	0.3 ± 0.2%	1.2 ± 0.6%	5.0 ± 2.1%	38.1 ± 7.8%	55.5 ± 10.3%
	1,000	0.2 ± 0.2%	0.9 ± 0.4%	3.7 ± 1.6%	32.4 ± 7.7%	62.8 ± 9.6%
	1,500	0.2 ± 0.1%	0.6 ± 0.3%	2.8 ± 1.3%	27.0 ± 7.7%	69.4 ± 9.2%
	2,000	0.1 ± 0.1%	0.5 ± 0.3%	2.1 ± 1.1%	22.0 ± 7.8%	75.2 ± 9.2%
	2,500	0.1 ± 0.1%	0.4 ± 0.3%	1.6 ± 1.0%	17.9 ± 7.9%	80.1 ± 9.2%
	3,000	0.1 ± 0.1%	0.3 ± 0.2%	1.3 ± 1.0%	14.4 ± 7.9%	83.9 ± 9.1%
	3,500	0.1 ± 0.1%	0.2 ± 0.2%	1.0 ± 0.9%	11.7 ± 7.8%	87.0 ± 8.9%
	4,000	0.1 ± 0.1%	0.2 ± 0.2%	0.8 ± 0.9%	9.5 ± 7.6%	89.4 ± 8.7%
	4,500	0.0 ± 0.1%	0.2 ± 0.2%	0.7 ± 0.9%	7.8 ± 7.4%	91.3 ± 8.5%
	5,000	0.0 ± 0.1%	0.1 ± 0.3%	0.5 ± 0.9%	6.5 ± 7.1%	92.8 ± 8.2%

*GLMM, generalized linear mixed model; IADL, instrumental activities of daily living; LEDD, levodopa equivalent daily dose; Pr(resp = i), probability that a patient will respond to any item of The Penn Parkinson’s Daily Activities Questionnaire-15 (PDAQ-15) with the response “i” where “i” represents difficulties in IADL and can take on values 0 = “cannot do,” 1 = “a lot,” 2 = “somewhat,” 3 = “a little,” and 4 = “none”; the numbers represent posterior predictions of the ordered-logit GLMM for a patient with an average cognitive performance (Dementia Rating Scale, DRS-2≈139) and level of depressive symptoms (Beck Depression Inventory, BDI-II≈10) described in the main text in a format mean ± standard deviation.*

## Discussion

This study examined IADL in a cohort of PD patients undergoing STN-DBS treatment by using pre-test (pre-surgery) and post-test (post-surgery) measurements. Regarding the complexity of DBS neurosurgical treatment, only IADL self-report and cognitive, depressive, and clinical correlates were included in the current research. In comparison to other studies that concentrated either on IADL predictors or the quantification of the degree of IADL deficits in PD-NC in comparison to PD-MCI (Rosenthal et al., 2010; Pirogovsky et al., 2013; Foster, 2014; Fellows and Schmitter-Edgecombe, 2019; Becker et al., 2020; Cholerton et al., 2020; Foster and Doty, 2021; Schmitter-Edgecombe et al., 2021; Becker et al., 2022), or comparison and development of specific methods and sensitive IADL items for PD (Brennan et al., 2016a,b; Fellows and Schmitter-Edgecombe, 2019; Sulzer et al., 2020; Schmitter-Edgecombe et al., 2021), whereas our study focused selectively on the comparison of IADL in PD before and after STN-DBS with a PD-specific questionnaire (Brennan et al., 2016a,b). We based our estimate of the post-surgery IADL change on an explicit causal model allowing for easier model criticism and derivation of proper covariates to include in our model (Pearl, 2009; McElreath, 2020).

Based on our model and data, the post-surgery IADL of PD patients improves compared to the pre-surgery level. At the same time, higher cognitive performance and higher LEDD are indicative of higher IADL both before and after STN-DBS surgery. More specifically, our analysis focused on the direct effects of all included predictors, in other words, the estimation of IADL post-surgery improvement was thus adjusted on LEDD, DRS-2, and BDI-II. While this approach allowed us to derive a more accurate estimate of the direct effect DBS can have on IADL performance in a cohort of implanted patients, this effect will be in the real-life clinical settings contaminated by DBS effects on other variables predictive of IADL change. Indeed, according to our results, the IADL declines when LEDD is decreased both before and after surgery, however, a decrease in LEDD is often a desirable outcome of DBS treatment (Molinuevo et al., 2000; Russmann et al., 2004). Surprisingly, there was a trend of an interaction between the time of assessment and LEDD indicating that higher LEDD may be more important for IADL improvement post-surgery than pre-surgery.

As a consequence of the above described putatively opposing effects of DBS and post-surgery LEDD reduction, medical professionals may want to carefully consider how much to reduce the LEDD after STN-DBS surgery in PD patients to avoid negative effects on IADL. In the current study, these considerations are quantitatively represented in Table 3 which can be used to guide decisions on how much to decrease the LEDD after STN-DBS surgery while avoiding adverse effects on IADL. For instance, based on Table 3, one can expect that a patient with pre-surgery LEDD of 2,500 mg will report no difficulties (response four) in IADL about 62% of the time while reporting little difficulties (response three) about 33% of the time. If a physician was to reduce this patient’s LEDD after the surgery to 1,000 mg, one can expect that patient’s IADL would remain similar to the pre-surgery level reporting no difficulties about 63% of the time and little difficulties about 33% of the time. However, if the LEDD was discontinued altogether, the expectation of IADL difficulties would increase to a level where one would expect the patient to report no problems only 48% of the time and little problems about 43% of the time. In this case, it would thus be advisable not to reduce LEDD below 1,000 mg if the patient wanted to avoid possible adverse effects on IADL. This finding can be considered as an example of the “masked” effect (McElreath, 2020) with LEDD playing a crucial role in modulating the effect of STN-DBS on IADLs.

The present study suffers from several limitations that must be clearly stated. First, we did not apply multiple IADL assessment methods (i.e., observed everyday activities, self- and informant-reports) which would show different facets of IADL (Fellows and Schmitter-Edgecombe, 2019; Schmitter-Edgecombe et al., 2021). However, it is questionable in our PD sample with PD-NC or PD-MCI in the early stages of cognitive decline if self-rating is not more sensitive to the impact of cognitive changes on IADL function than informant reports (Cholerton et al., 2020). Second, our research is not longitudinal and we are not able to trace long-lasting changes in IADL due to STN-DBS. Third, we do not report data on individual stimulation volumes and functional zones of the STN and their contribution to IADL changes (Tödt et al., 2022). Fourth, a modest sample size regarding DBS research and the apparent lack of cognitive decline after STN-DBS surgery in our sample might have prevented us from observing any significant interaction between the time of assessment and cognitive performance. Fifth, an important limitation of the study is the lack of a control group. The influence of LEDD and global cognition on the improvement of IADL at 1 year of follow-up could be independent of the effect of DBS on motor symptoms. Such comparison cannot be performed due to a lack of a control group. However, based on previous research, the neurostimulation, as compared with medication alone, caused greater improvements from baseline to 6 months and DBS and levodopa have a differential effect on brain motor activity in PD (Deuschl et al., 2006; Mueller et al., 2020).

The current study shows a clear beneficial STN-DBS-induced change in IADL approximately 1-year perspective after the operation. Importantly, we show the IADL post-surgery improvement is related also to LEDD post-surgery medication dose that should not decrease under a certain limit to maintain the positive IADL effect of the surgery. Based on our study, STN-DBS seems as a cognitively safe procedure for the treatment of motor symptoms in PD 1 year after the surgery, however, a lower global cognitive functioning in the pre-surgery phase is associated with lower IADL functioning before and after the operation. Understanding the role of IADL functioning in PD in the pre-surgery phase may help identify those at risk for everyday activities and possibly help to improve interventions to promote functional independence after the electrode implantation.

## Data Availability Statement

The original contributions presented in this study are included in the article/Supplementary Material, further inquiries can be directed to the corresponding author.

## Ethics Statement

The studies involving human participants were reviewed and approved by the Ethics Committee of the General University Hospital in Prague, Czechia. The patients/participants provided their written informed consent to participate in this study.

## Author Contributions

OB: conceptualization, data curation, investigation, methodology, supervision, writing—original draft and review and editing. JM: conceptualization, data curation, investigation, formal analysis, methodology, software, visualization, writing—original draft and review and editing. FR, FH, AF, TU, and DU: investigation and writing—review and editing. ER: conceptualization, funding acquisition, investigation, resources, and writing—review and editing. RJ: conceptualization, data acquisition and curation, funding acquisition, investigation, resources, supervision, and writing—review and editing. All authors contributed to the article and approved the submitted version.

## Conflict of Interest

The authors declare that the research was conducted in the absence of any commercial or financial relationships that could be construed as a potential conflict of interest.

## Publisher’s Note

All claims expressed in this article are solely those of the authors and do not necessarily represent those of their affiliated organizations, or those of the publisher, the editors and the reviewers. Any product that may be evaluated in this article, or claim that may be made by its manufacturer, is not guaranteed or endorsed by the publisher.

## References

[B1] Altieri M. Garramone F. Santangelo G. (2021). Functional autonomy in dementia of the Alzheimer’s type, mild cognitive impairment, and healthy aging: a meta-analysis. *Neurol. Sci.* 42 1773–1783. 10.1007/s10072-021-05142-0 33738665

[B2] Beck A. T. Steer R. A. Brown G. (1996). *Manual For The Beck Depression Inventory-II.* San Antonio, TX: Pearson.

[B3] Becker S. Bäumer A. Maetzler W. Nussbaum S. Timmers M. Van Nueten L. (2020). Assessment of cognitive-driven activity of daily living impairment in non-demented Parkinson’s patients. *J. Neuropsychol.* 14 69–84. 10.1111/jnp.12173 30320954

[B4] Becker S. Pauly C. Lawton M. Hipp G. Bowring F. Sulzer P. (2022). Quantifying activities of daily living impairment in Parkinson’s disease using the Functional Activities Questionnaire. *Neurol. Sci.* 43 1047–1054. 10.1007/s10072-021-05365-1 34109514 PMC8789696

[B5] Bezdicek O. Michalec J. Nikolai T. Havrankova P. Roth J. Jech R. (2015). Clinical validity of the Mattis Dementia Rating Scale in differentiating mild cognitive impairment in Parkinson’s disease and normative data. *Dement. Geriatr. Cogn. Disord.* 39 303–311. 10.1159/000375365 25792240

[B6] Bezdicek O. Nikolai T. Michalec J. Růžička F. Havránková P. Roth J. (2016). The diagnostic accuracy of parkinson’s disease mild cognitive impairment battery using the movement disorder society task force criteria. *Mov. Disord. Clin. Pract.* 4 237–244. 10.1002/mdc3.12391 30363396 PMC6174468

[B7] Bezdicek O. Sulc Z. Nikolai T. Stepankova H. Kopecek M. Jech R. (2017). A parsimonious scoring and normative calculator for the Parkinson’s disease mild cognitive impairment battery. *Clin. Neuropsychol.* 31 1231–1247. 10.1080/13854046.2017.1293161 28276860

[B8] Bratsos S. Karponis D. Saleh S. N. (2018). Efficacy and Safety of Deep Brain Stimulation in the Treatment of Parkinson’s Disease: a systematic review and meta-analysis of randomized controlled trials. *Cureus* 10:e3474. 10.7759/cureus.3474 30648026 PMC6318091

[B9] Brennan L. Siderowf A. Rubright J. D. Rick J. Dahodwala N. Duda J. E. (2016a). Development and initial testing of the penn Parkinson’s daily activities questionnaire. *Mov Disord* 31 126–134. 10.1002/mds.26339 26249849 PMC4724261

[B10] Brennan L. Siderowf A. Rubright J. D. Rick J. Dahodwala N. Duda J. E. (2016b). The penn Parkinson’s daily activities questionnaire-15: psychometric properties of a brief assessment of cognitive instrumental activities of daily living in Parkinson’s disease. *Parkinsonism Relat. Disord.* 25 21–26. 10.1016/j.parkreldis.2016.02.020 26923524 PMC4818172

[B11] Bronnick K. Ehrt U. Emre M. De Deyn P. P. Wesnes K. Tekin S. (2006). Attentional deficits affect activities of daily living in dementia-associated with Parkinson’s disease. *J. Neurol. Neurosurg. Psychiatry* 77 1136–1142. 10.1136/jnnp.2006.093146 16801351 PMC2077544

[B12] Bronstein J. M. Tagliati M. Alterman R. L. Lozano A. M. Volkmann J. Stefani A. (2011). Deep brain stimulation for Parkinson disease: an expert consensus and review of key issues. *Arch. Neurol.* 68:165. 10.1001/archneurol.2010.260 20937936 PMC4523130

[B13] Bürkner P.-C. Vuorre M. (2019). Ordinal regression models in psychology: a tutorial. *Adv. Methods Pract. Psychol. Sci.* 2 77–101.

[B14] Cahn D. A. Sullivan E. V. Shear P. K. Pfefferbaum A. Heit G. Silverberg G. (1998). Differential contributions of cognitive and motor component processes to physical and instrumental activities of daily living in Parkinson’s disease. *Arch. Clin. Neuropsychol.* 13 575–583. 14590618

[B15] Cholerton B. Poston K. L. Tian L. Quinn J. F. Chung K. A. Hiller A. L. (2020). Participant and study partner reported impact of cognition on functional activities in parkinson’s disease. *Mov. Disord. Clin. Pract.* 7 61–69. 10.1002/mdc3.12870 31970213 PMC6962683

[B16] Christ J. B. Fruhmann Berger M. Riedl E. Prakash D. Csoti I. Molt W. (2013). How precise are activities of daily living scales for the diagnosis of Parkinson’s disease dementia? A pilot study. *Parkinsonism Relat. Disord.* 19 371–374. 10.1016/j.parkreldis.2012.11.004 23231974

[B17] Ciharova M. Cígler H. Dostálová V. Šivicová G. Bezdicek O. (2020). Beck depression inventory, second edition, Czech version: demographic correlates, factor structure and comparison with foreign data. *Int. J. Psychiatry Clin. Pract.* 24 371–379. 10.1080/13651501.2020.1775854 32552177

[B18] Deuschl G. Schade-Brittinger C. Krack P. Volkmann J. Schäfer H. Bötzel K. (2006). A randomized trial of deep-brain stimulation for Parkinson’s disease. *N. Engl. J. Med.* 355 896–908. 10.1056/NEJMoa060281 16943402

[B19] Emre M. Aarsland D. Brown R. Burn D. J. Duyckaerts C. Mizuno Y. (2007). Clinical diagnostic criteria for dementia associated with Parkinson’s disease. *Mov. Disord.* 22 1689–1707; quiz1837. 10.1002/mds.21507 17542011

[B20] Fellows R. P. Schmitter-Edgecombe M. (2019). Multimethod assessment of everyday functioning and memory abilities in Parkinson’s disease. *Neuropsychology* 33 169–177. 10.1037/neu0000505 30451512

[B21] Foley J. A. Foltynie T. Limousin P. Cipolotti L. (2018). Standardised neuropsychological assessment for the selection of patients undergoing DBS for Parkinson’s Disease. *Parkinsons Dis.* 2018:4328371. 10.1155/2018/4328371 29971141 PMC6009029

[B22] Foster E. R. (2014). Instrumental activities of daily living performance among people with Parkinson’s disease without dementia. *Am. J. Occup. Ther.* 68 353–362. 10.5014/ajot.2014.010330 24797199 PMC4011459

[B23] Foster E. R. Doty T. (2021). Cognitive correlates of instrumental activities of daily living performance in parkinson disease without dementia. *Arch. Rehabil. Res. Clin. Transl.* 3:100138. 10.1016/j.arrct.2021.100138 34589688 PMC8463453

[B24] Giovannetti T. Britnell P. Brennan L. Siderowf A. Grossman M. Libon D. J. (2012). Everyday action impairment in Parkinson’s disease dementia. *J. Int. Neuropsychol. Soc.* 18 787–798. 10.1017/S135561771200046X 22621995 PMC3648638

[B25] Gorecka-Mazur A. Furgala A. Krygowska-Wajs A. Pietraszko W. Kwinta B. Gil K. (2019). Activities of daily living and their relationship to health-related quality of life in patients with parkinson disease after subthalamic nucleus deep brain stimulation. *World Neurosurg.* 125 e552–e562. 10.1016/j.wneu.2019.01.132 30716489

[B26] Hariz G.-M. Forsgren L. (2011). Activities of daily living and quality of life in persons with newly diagnosed Parkinson’s disease according to subtype of disease, and in comparison to healthy controls. *Acta Neurol. Scand.* 123 20–27. 10.1111/j.1600-0404.2010.01344.x 20199514

[B27] Hentz J. G. Mehta S. H. Shill H. A. Driver-Dunckley E. Beach T. G. Adler C. H. (2015). Simplified conversion method for unified Parkinson’s disease rating scale motor examinations. *Mov. Disord.* 30 1967–1970. 10.1002/mds.26435 26779608 PMC4717915

[B28] Hoogland J. Boel J. A. De Bie R. M. A. Geskus R. B. Schmand B. A. Dalrymple-Alford J. C. (2017). Mild cognitive impairment as a risk factor for Parkinson’s disease dementia. *Mov. Disord.* 32 1056–1065. 10.1002/mds.27002 28605056

[B29] Hughes A. J. Daniel S. E. Kilford L. Lees A. J. (1992). Accuracy of clinical diagnosis of idiopathic Parkinson’s disease: a clinico-pathological study of 100 cases. *J. Neurol. Neurosurg. Psychiatry* 55 181–184. 10.1136/jnnp.55.3.181 1564476 PMC1014720

[B30] Jech R. Mueller K. Urgošík D. Sieger T. Holiga Š Růžička F. (2012). The Subthalamic microlesion story in Parkinson’s disease: electrode insertion-related motor improvement with relative cortico-subcortical hypoactivation in fMRI. *PLoS One* 7:e49056. 10.1371/journal.pone.0049056 23145068 PMC3492182

[B31] Jech R. Ruzicka E. Urgosik D. Serranova T. Volfova M. Novakova O. (2006). Deep brain stimulation of the subthalamic nucleus affects resting EEG and visual evoked potentials in Parkinson’s disease. *Clin. Neurophysiol.* 117 1017–1028. 10.1016/j.clinph.2006.01.009 16516544

[B32] Jiang J.-L. Chen S.-Y. Hsieh T.-C. Lee C.-W. Lin S.-H. Tsai S.-T. (2015). Different effectiveness of subthalamic deep brain stimulation in Parkinson’s disease: a comparative cohort study at 1 year and 5 years. *J. Formos. Med. Assoc.* 114 835–841. 10.1016/j.jfma.2013.09.006 24103710

[B33] Jurica P. J. Leitten C. L. Mattis S. (2001). *Dementia Rating Scale-2 (DRS-2) Professional Manual.* Lutz, FL: Psychological Assessment Resources.

[B34] Kleiner-Fisman G. Stern M. B. Fisman D. N. (2010). Health-related quality of life in parkinson disease: correlation between health utilities index iii and unified parkinson’s disease rating scale (UPDRS) in U.S. male veterans. *Health Qual. Life Outcomes* 8:91. 10.1186/1477-7525-8-91 20799993 PMC2939643

[B35] Kubu C. S. (2018). The role of a neuropsychologist on a movement disorders deep brain stimulation team. *Arch. Clin. Neuropsychol.* 33 365–374. 10.1093/arclin/acx130 29718080 PMC7328472

[B36] Lawson R. A. Williams-Gray C. H. Camacho M. Duncan G. W. Khoo T. K. Breen D. P. (2021). Which neuropsychological tests? Predicting cognitive decline and dementia in Parkinson’s Disease in the ICICLE-PD cohort. *J. Parkinsons Dis.* 11 1297–1308. 10.3233/JPD-212581 34024781 PMC8461722

[B37] Liddell T. M. Kruschke J. K. (2018). Analyzing ordinal data with metric models: what could possibly go wrong? *J. Exp. Soc. Psychol.* 79 328–348.

[B38] Litvan I. Goldman J. G. Tröster A. I. Schmand B. A. Weintraub D. Petersen R. C. (2012). Diagnostic criteria for mild cognitive impairment in Parkinson’s disease: movement disorder society task force guidelines. *Mov. Disord.* 27 349–356. 10.1002/mds.24893 22275317 PMC3641655

[B39] Martin R. C. Triebel K. L. Kennedy R. E. Nicholas A. P. Watts R. L. Stover N. P. (2013). Impaired financial abilities in Parkinson’s disease patients with mild cognitive impairment and dementia. *Parkinsonism Relat. Disord.* 19 986–990. 10.1016/j.parkreldis.2013.06.017 23899743 PMC4652594

[B40] McElreath R. (2020). *Statistical Rethinking: A Bayesian Course With Examples in R and Stan.* London: Chapman and Hall/CRC.

[B41] Molinuevo J. L. Valldeoriola F. Tolosa E. Rumià J. Valls-Solé J. Roldán H. (2000). Levodopa withdrawal after bilateral subthalamic nucleus stimulation in advanced parkinson disease. *Arch. Neurol.* 57 983–988. 10.1001/archneur.57.7.983 10891980

[B42] Moro E. Lang A. E. (2006). Criteria for deep-brain stimulation in Parkinson’s disease: review and analysis. *Exp. Rev. Neurother.* 6 1695–1705. 10.1586/14737175.6.11.1695 17144783

[B43] Mueller K. Urgošík D. Ballarini T. Holiga Š Möller H. E. Růžička F. (2020). Differential effects of deep brain stimulation and levodopa on brain activity in Parkinson’s disease. *Brain Commun.* 2:fcaa005. 10.1093/braincomms/fcaa005 32954278 PMC7425344

[B44] Odekerken V. J. J. Van Laar T. Staal M. J. Mosch A. Hoffmann C. F. E. Nijssen P. C. G. (2013). *Subthalamic nucleus* versus *Globus pallidus* bilateral deep brain stimulation for advanced Parkinson’s disease (NSTAPS study): a randomised controlled trial. *Lancet Neurol.* 12 37–44. 10.1016/S1474-4422(12)70264-8 23168021

[B45] Okun M. S. (2014). Deep-brain stimulation–entering the era of human neural-network modulation. *N. Engl. J. Med.* 371 1369–1373. 10.1056/NEJMp1408779 25197963

[B46] Pearl J. (2009). *Causality: Models, Reasoning, and Inference.* Cambridge: Cambridge University Press.

[B47] Perlmutter J. S. Mink J. W. (2006). Deep brain stimulation. *Ann. Rev. Neurosci.* 29 229–257. 16776585 10.1146/annurev.neuro.29.051605.112824 PMC4518728

[B48] Pirogovsky E. Martinez-Hannon M. Schiehser D. M. Lessig S. L. Song D. D. Litvan I. (2013). Predictors of performance-based measures of instrumental activities of daily living in nondemented patients with Parkinson’s disease. *J. Clin. Exp. Neuropsychol.* 35 926–933. 10.1080/13803395.2013.838940 24074137

[B49] Pirogovsky E. Schiehser D. M. Obtera K. M. Burke M. M. Lessig S. L. Song D. D. (2014). Instrumental activities of daily living are impaired in Parkinson’s disease patients with mild cognitive impairment. *Neuropsychology* 28 229–237. 10.1037/neu0000045 24417192

[B50] R Core Team (2021). *R: A Language and Environment for Statistical Computing.* Vienna: R Foundation for Statistical Computing.

[B51] Rosenthal E. Brennan L. Xie S. Hurtig H. Milber J. Weintraub D. (2010). Association between cognition and function in patients with Parkinson disease with and without dementia. *Mov. Disord.* 25 1170–1176. 10.1002/mds.23073 20310053 PMC2963089

[B52] Russmann H. Ghika J. Combrement P. Villemure J. G. Bogousslavsky J. Burkhard P. R. (2004). L-Dopa-induced dyskinesia improvement after STN-DBS depends upon medication reduction. *Neurology* 63 153–155. 10.1212/01.wnl.0000131910.72829.9d 15249627

[B53] Schmitter-Edgecombe M. McAlister C. Greeley D. (2021). A comparison of functional abilities in individuals with mild cognitive impairment and Parkinson’s disease with mild cognitive impairment using multiple assessment methods. *J. Int. Neuropsychol. Soc.* 1–12. 10.1017/S1355617721001077 [Epub ahead of print]. 34486508 PMC8898320

[B54] Shulman L. M. Armstrong M. Ellis T. Gruber-Baldini A. Horak F. Nieuwboer A. (2016). Disability rating scales in parkinson’s disease: critique and recommendations. *Mov. Disord.* 31 1455–1465. 10.1002/mds.26649 27193358

[B55] Soh S.-E. Mcginley J. L. Watts J. J. Iansek R. Murphy A. T. Menz H. B. (2013). Determinants of health-related quality of life in people with Parkinson’s disease: a path analysis. *Qual. Life Res.* 22 1543–1553. 10.1007/s11136-012-0289-1 23070750

[B56] Stan Development Team (2021). *Stan Modeling Language User’s Guide and Reference Manual (Version 2.21.0)*. Available online at: http://mc-stan.org/

[B57] Sulzer P. Liebig L. Csoti I. Graessel E. Wurster I. Berg D. (2020). A time-efficient screening tool for activities of daily living functions in Parkinson’s disease dementia. *J. Clin. Exp. Neuropsychol.* 42 867–879. 10.1080/13803395.2020.1825634 33043797

[B58] Tödt I. Al-Fatly B. Granert O. Kühn A. A. Krack P. Rau J. (2022). The contribution of subthalamic nucleus deep brain stimulation to the improvement in motor functions and quality of life. *Mov. Disord.* 37 291–301. 10.1002/mds.28952 35112384

[B59] Tomlinson C. L. Stowe R. Patel S. Rick C. Gray R. Clarke C. E. (2010). Systematic review of levodopa dose equivalency reporting in Parkinson’s disease. *Mov. Disord.* 25 2649–2653. 10.1002/mds.23429 21069833

[B60] Urgosik D. Jech R. Ruzicka E. Ruzicka F. (2011). Deep brain stimulation in movement disorders: a prague-center experience. *Cas Lek Cesk* 150 223–228. 21634199

[B61] Young T. L. Granic A. Yu Chen T. Haley C. B. Edwards J. D. (2010). Everyday reasoning abilities in persons with Parkinson’s disease. *Mov. Disord.* 25 2756–2761. 10.1002/mds.23379 20939079 PMC3003758

