# Supplementary material for: The Instrumental Activities of Daily Living in Parkinson’s Disease Patients Treated by Subthalamic Deep Brain Stimulation
Source: Front Aging Neurosci. 2022 Jun 17;14:886491. doi: 10.3389/fnagi.2022.886491 (PMC9247575; doi:10.3389/fnagi.2022.886491)
Supplement: Supplementary file 1 [file Image_1.pdf]

Supplementary Material

Supplementary Figure 1. Patients' responses to each PDAQ-15 item before and after the STN-DBS surgery

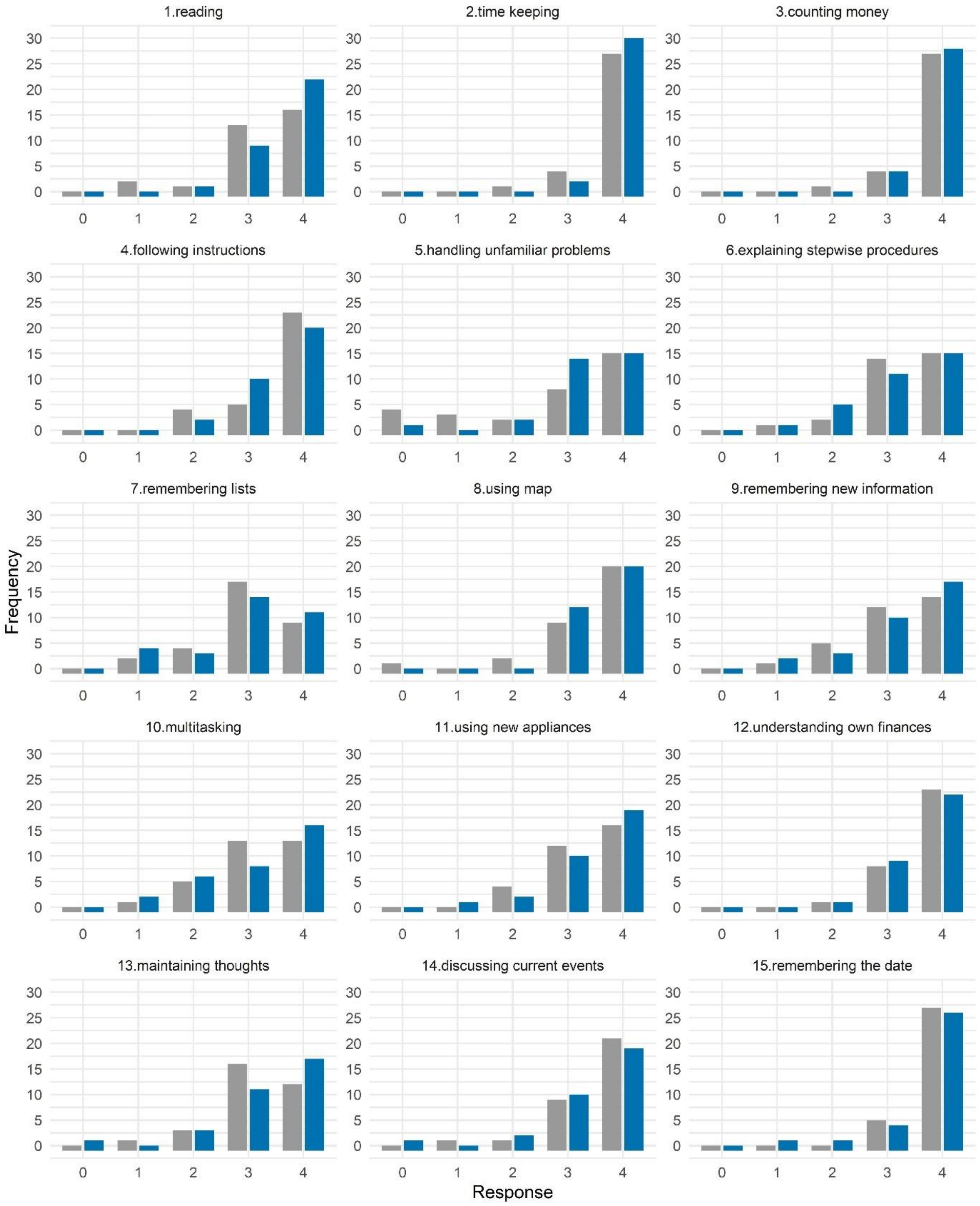

Note. Grey colour = pre-surgery; blue = post-surgery. PDAQ-15 = 15 items included in the Penn Parkinson's Daily Activities Questionnair-15 (range 0–4 represents difficulty with single instrumental activities of daily living; None (4); A Little (3); Somewhat (2); A Lot (1); Cannot Do (0)).
